# Coronavirus Disease Pandemic Is a Real Challenge for Brazil

**DOI:** 10.3389/fpubh.2020.00268

**Published:** 2020-06-05

**Authors:** Ana Cristina Simões e Silva, Eduardo A. Oliveira, Hercílio Martelli

**Affiliations:** ^1^Department of Pediatrics, Faculty of Medicine, Federal University of Minas Gerais (UFMG), Belo Horizonte, Brazil; ^2^Program Health Sciences - Primary Health Care, State University of Montes Claros (UNIMONTES), Montes Claros, Brazil

**Keywords:** COVID-19, public health, Brazil, social isolation, pandemic

In December 2019, a cluster of pneumonia cases of unknown etiology was reported in Wuhan, China (1). On January 7, a novel coronavirus was identified from the throat swab sample of a patient (2), and by January 2020, the virus had been isolated and sequenced (3). The new virus was subsequently named SARS-CoV-2/human/Wuhan/X1/2019 (SARS-CoV-2) (4). On March 11, 2020, the WHO announced that the disease caused by SARS-CoV-2, designated COVID-19, should be considered a global pandemic (5). By May 03, 2020, there were already 3,349,786 confirmed cases of contamination and 238,628 deaths throughout almost the whole world (6). This first pandemic of the twenty-first century places unprecedented pressure on societies and healthcare systems around the world. As pointed out by Jones in a recent commentary, “*a history of epidemics offers considerable advice, but only if people know the history and respond with wisdom*” (7).

Approximately 56 days after the first case reported in China, on February 26, Brazil officially registered its first patient with COVID-19: a 61-year-old man living in São Paulo who had recently returned from a trip to Italy. Twenty days after the first reported case (March 17, 2020), Brazil registered the first death by COVID-19 in a 62-year-old man with diabetes and heart disease (8). On March 30, 2020, Brazil recorded 4,470 confirmed cases and 159 deaths. By May 25, 2020, Brazil had already experienced 363,211 confirmed cases and 22,666 deaths by COVID-19 (https://covid.saude.gov.br/). However, it should be noted that these numbers underestimate the real depth of the pandemic in Brazil. This is because, to date, capacity for a massive surge in laboratory testing has not been enabled in our country (9). In this respect, to decentralize the diagnosis of coronavirus, institutes linked to the Ministry of Health have become responsible for training 27 Central Public Health Laboratories on testing, starting in February 2020. Since March 18, Central Public Health Laboratories from 26 states and the Federal District have been considered able to perform tests for coronavirus. Nevertheless, in this regard, to date, the country is far below the optimal number of tests for COVID-19, as there are not enough tests to achieve a reliable panorama of the real number of cases. Currently the rate in Brazil is only 14.5 tests/million as compared with the rates of >70 in Italy and the UK, for example.

The distribution of the resident population according to age group shows a downward trend in the proportion of people <30 years old along with an increase in the proportion of older people. In 2012, people below 30 years old represented 47.6% of the population. This proportion decreased to 42.9% in 2018, while the proportion over 30 years old increased to 57.1% (10). Moreover, chronic diseases, especially systemic arterial hypertension and diabetes mellitus, and their related morbidity and mortality are currently a prevalent public health issue. Data from the Ministry of Health show that the prevalence of hypertension and diabetes among Brazilian adults aged 35 and older was 24.3 and 11.7%, respectively. The rates are higher in people aged over 65, in whom the prevalence rises to 54.9 % for hypertension and 19.3% for diabetes. With the rapid spread of COVID-19, by the end of March, the main Brazilian states had adopted a series of social distancing measures. These included recommending that older adults and individuals with chronic medical conditions stay at home as much as possible, canceling mass events, closing schools, universities, and workplaces, and maintaining only essential services (8). Furthermore, the Ministry of Health is hiring 5,811 emergency physicians, particularly in poorer cities and indigenous villages, to work to control disease spread.

The collapse of healthcare systems is the major concern for most countries hit by the pandemic, especially low- and middle-income countries, such as Brazil. For instance, among the confirmed cases in China, 18.5% were considered severe, and 25.3% of those required intensive care. Among 4,103 COVID-19 patients in New York, 1,999 (48.7%) were hospitalized, and 445 patients (10.8%) required mechanical ventilation (11). Therefore, a critical aspect of the COVID-19 pandemic is healthcare system capacity. Since 1989, Brazil has established a universal public health system (SUS, *Sistema Único de Saúde*) that, in this current pandemic scenario, allowed a coordinated response among the diverse federation units (12). However, our capacity to deal with critical cases is limited and very heterogeneous across the 26 states. In Brazil, the number of intensive care units (ICUs) through February 2020 amounted to 36,939 beds, according to the *Cadastro Nacional de Estabelecimentos de Saúde* (CNES), with a historical occupancy of not <85%, which yields an ~5500 free ICU beds. The global European number of ICUs per 100,000 inhabitants is ~10, with the US leading the world with a ratio of 34.7:100,000; both, however, are far below what is expected to be needed as the number of infections approaches its peak (13).

In the absence of any efficient treatment and/or vaccine to impede the fast spread of the disease, many public policies and governmental strategies, termed non-pharmaceutical interventions (NPIs), have been used amid the epidemic/pandemic situation. Currently, many such public health measures involve reducing social contact in the population and, consequently, the transmission rate of the virus, alleviating the pressure on the health system and providing time for auxiliary measures to be put in place (expansion of the system, creation of military hospitals, and so on). In this regard, another critical aspect is the difference in population adherence to social isolation measures in the different cities and states of the country (14).

It is worth mentioning that all of these measures have critical socioeconomic and ethical implications because they severely interfere with the outflow of industrial products and commodities, reduce spontaneous social aggregations, and so on. Therefore, to lift these drastic measures after the control of the initial wave, which is expected to demonstrate exponential growth in the number of confirmed cases, the WHO has recommended that isolating, testing, and treating every suspected case and tracing every contact must form the backbone for every country's response. This is the best hope for preventing widespread community transmission. Most countries with sporadic cases or clusters of cases are still in a position to do this. Many countries are following the WHO recommendations and finding solutions to increase their ability to implement the full package of measures.

In summary, the Brazilian challenge is not only to stop the spread of COVID-19 but also to find agreement between political leaders, scientific societies, and the general population. The Brazilian scientific community and healthcare workers are working hard to provide support for political health measures to address COVID-19 (15, 16). Hopefully, this pandemic may be an opportunity for political leaders and the general population to clearly comprehend the pivotal importance of science and the public health system in their daily lives. In this regard, a recent editorial highlighted the difficulty of imagining a world that has not been permanently changed by COVID-19 (17). Thorp, the editor of Science Magazine, considered that the success of the world's scientists, along with strong political and social leadership, will determine which scenarios unfold, so it is time to focus on what we can all do to help (17, 18). Thus, the only way to deal with pandemics is with solidarity and cooperative measures from political leaders, scientists, healthcare providers, and the general population.

## Author Contributions

AS, EO, and HM collected data, wrote the paper, and aprroved the final version. All authors contributed to the article and approved the submitted version.

## Conflict of Interest

The authors declare that the research was conducted in the absence of any commercial or financial relationships that could be construed as a potential conflict of interest.
